# Early results of a natural experiment evaluating the effects of a local minimum wage policy on the diet-related health of low-wage workers, 2018–2020

**DOI:** 10.1017/S1368980023001520

**Published:** 2023-11

**Authors:** Caitlin E Caspi, Maria Fernanda Gombi-Vaca, Julian Wolfson, Lisa J Harnack, Molly De Marco, Rebekah Pratt, Thomas Durfee, Samuel L. Myers

**Affiliations:** 1 Rudd Center for Food Policy and Health, University of Connecticut, 1 Constitution Plaza, Hartford, CT 061032, USA; 2 Department of Allied Health Sciences, University of Connecticut, 358 Mansfield Dr., Storrs, CT 06269, USA; 3 Division of Biostatistics, School of Public Health, University of Minnesota, A460 Mayo Building MMC 303, 425 Delaware St. SE, Minneapolis, MN, USA; 4 Division of Epidemiology and Community Health, Suite 300, University of Minnesota, 1300 South 2nd St, Minneapolis, MN, USA; 5 Center for Health Promotion & Disease Prevention, University of North Carolina at Chapel Hill, 1700 M.L.K. Jr Blvd #7426, Chapel Hill, NC 27514, USA; 6 Department of Nutrition, Gillings School of Global Public Health, UNC-CH, 135 Dauer Dr, Chapel Hill, NC 27599, USA; 7 Department of Family Medicine and Community Health, University of Minnesota, 717 Delaware St. se, Minneapolis, MN 55445, USA; 8 The Roy Wilkins Center for Human Relations and Social Justice, Hubert H. Humphrey School of Public Affairs, University of Minnesota, 270 Humphrey Center, 301 19th Avenue South, Minneapolis, MN, USA; 9 Department of Applied Economics, University of Minnesota, 231 Ruttan Hall, 1994 Buford Avenue, St. Paul, MN, USA

**Keywords:** Policy evaluation, Social determinants of health, Minimum wage, Food insecurity, COVID-19

## Abstract

**Objective:**

The current study presents results of a midpoint analysis of an ongoing natural experiment evaluating the diet-related effects of the Minneapolis Minimum Wage Ordinance, which incrementally increases the minimum wage to $15/h.

**Design:**

A difference-in-difference (DiD) analysis of measures collected among low-wage workers in two U.S. cities (one city with a wage increase policy and one comparison city). Measures included employment-related variables (hourly wage, hours worked and non-employment assessed by survey questions with wages verified by paystubs), BMI measured by study scales and stadiometers and diet-related mediators (food insecurity, Supplemental Nutrition Assistance Program (SNAP) participation and daily servings of fruits and vegetables, whole-grain rich foods and foods high in added sugars measured by survey questions).

**Setting:**

Minneapolis, Minnesota and Raleigh, North Carolina.

**Participants:**

A cohort of 580 low-wage workers (268 in Minneapolis and 312 in Raleigh) who completed three annual study visits between 2018 and 2020.

**Results:**

In DiD models adjusted for time-varying and non-time-varying confounders, there were no statistically significant differences in variables of interest in Minneapolis compared with Raleigh. Trends across both cities were evident, showing a steady increase in hourly wage, stable BMI, an overall decrease in food insecurity and non-linear trends in employment, hours worked, SNAP participation and dietary outcomes.

**Conclusion:**

There was no evidence of a beneficial or adverse effect of the Minimum Wage Ordinance on health-related variables during a period of economic and social change. The COVID-19 pandemic and other contextual factors likely contributed to the observed trends in both cities.

In the USA, diet quality is socially patterned by race/ethnicity and socio-economic status. Adults minoritised as Black or Hispanic and those with a lower income disproportionately carry the disease burden associated with poor diet^(1,2)^. An array of food policy actions have the potential to promote a healthier diet quality in the USA, including point-of-purchase nutrition-labelling, strengthening nutrition standards for federal food programs and restricting targeted marketing of unhealthy food and beverages, among others^(3)^. However, non-food policies that address the social determinants of health^(4)^ also have the potential to address disparities in diet quality. Broadly, policies that promote economic stability can offer access to health promoting resources^(5,6)^ that support a healthy diet. For example, raising the minimum wage could address socio-economic disparities in diet-related outcomes by promoting food security, increasing purchasing power for more expensive healthy foods like fruits and vegetables and reducing consumption of inexpensive energy-dense foods^(7)^.

Current evidence is inconclusive for the effects of increasing the minimum wage on the outcomes of diet quality and BMI. Several studies have reported that an increase in minimum wage is associated with a decrease in BMI^(8–10)^; however, Andreyeva and Ukert found the opposite^(11)^. Meanwhile, there is conflicting evidence of the effect of increasing minimum wage on fruit and vegetable consumption^(8,12,13)^. Most studies examining the relationship between wages and diet-related outcomes have used proxy measures like education status to approximate the likelihood of being affected by area-level minimum wage increases, and/or use annual income as a proxy for wages without regard to hours or weeks worked^(5,14)^; most existing studies also use self-reported weight outcomes, which may be biased towards underreporting weight and overreporting height^(15)^.

New opportunities to evaluate the health-related effects of minimum wage policies have emerged as local and state minimum wage increases have been increasingly implemented^(16)^. The WAGE$ study follows low-wage workers in two cities to evaluate the diet-related effects of a phased-implementation of a $15 an hour local minimum wage ordinance. Workers likely to be affected by the ordinance in Minneapolis, Minnesota (MN), and those in a comparison city (Raleigh, North Carolina (NC)), were enrolled in the study in 2018 and are followed annually through 2022. In this time period, the minimum wage is increasing from $10 to $15 for large businesses and from $7·75 to $13·50 for small businesses in Minneapolis. The aims of the study are to test the effect of the minimum wage ordinance on change in BMI and other nutrition-related outcomes. The current report presents a DiD analysis after three annual visits of changes in job and diet-related factors among low-wage workers in both cities from 2018–2020. In 2020, the minimum wage had risen to $13·25 for workers of employers of greater than 100 employees and $11·75 for workers of smaller employers in Minneapolis.

The study was designed and initiated before the start of the COVID-19 pandemic. Between February and May of 2020, more than 14 million U.S. workers lost their jobs^(17)^, food insufficiency soared, particularly among non-Hispanic Black households and households with children^(18)^, and supply chains and business closures created disruption in food access for many^(19)^. At the same time, a suite of federal COVID-19 relief measures to promote economic security were enacted, which included a major U.S. Department of Agriculture (USDA) expansion of cash assistance benefit programs. The current analysis is based on two pre-COVID-19 measurement time points, as well as one time point that includes the unique social, economic and health circumstances of 2020. It is within this context that we interpret our results.

## Methods

### Conceptual model

We suggest that a minimum wage increase will improve health-promoting dietary behaviours and BMI^(20)^. In particular, we test whether diet-related mediators improve in the context of a minimum wage increase, including reduced food insecurity^(21)^ and improved diet quality^(9)^. The role of SNAP participation in mediating wages and diet-related outcomes is likely to be complex, but we expect that any SNAP benefit reductions are likely to be more than offset by the increase in wages^(22)^, with a net decrease in food insecurity. Our conceptual model also includes demographic, household and workforce factors and that the broader policy context that could enhance or diminish the effects of the minimum wage policy.

### Policy overview: the Minneapolis minimum wage ordinance

On June 30, 2017, the Minneapolis City Council passed the Minimum Wage Ordinance. The ordinance incrementally increases the minimum wage to $15 an hour. Employees are covered by the ordinance for time worked within the geographic boundaries of the city of Minneapolis if at least 2 h a week are worked. The ordinance does not apply to federal/state employees. Employers cannot apply tips to the minimum wage. In Raleigh, NC, the minimum wage was the federal minimum wage of $7·25 for non-tipped workers ($2·13 an hour for tipped workers).

### Study design

Participants were recruited from the Minneapolis and Raleigh communities in 2018. At annual appointments, participants completed an online survey, provided their paystub from all employers and conducted anthropometric height and weight measures. Appointments occurred in person at T1 (February 2018–October 2018) and T2 (July 2019–January 2020). Due to the COVID-19 pandemic, data collection occurred remotely at T3 (July 2020–January 2021) to mitigate the risk of COVID-19 to both research staff and participants.

### Selection of a comparison site

Complete details about the selection of the comparison site have been previously published^(23)^. Briefly, during the selection process, we first limited the possibilities to cities of similar size (within 50 % of the total Minneapolis population) located in states with a minimum wage preemption law to decrease the likelihood of a minimum wage policy change in the comparison city. We compared key demographics of each city and Minneapolis, including median household income, four racial/ethnic categories, poverty, percent foreign born, percent with greater than a high-school degree, employment rate, total businesses and median rent. A good match on each demographic was defined as a value within 25 % of Minneapolis. Raleigh matched on all demographics of interest except percent poverty (within 27 %) and percent Black (within 57 %). A better match for percent Black would have resulted in other tradeoffs. For example, selecting Arlington, TX, would have resulted in a match on percent Black, but a mismatch on percent Hispanic, percent foreign-born and education. We also ruled out violations of the parallel trends assumption for BMI over the previous 10-year period using Behavioral Risk Factor Surveillance System SMART data, compared the obesity rate, other cost of living measures and common industries across cities and ruled out differing trends in the economic trends in relevant industries in both metropolitan areas.

### Participant eligibility and recruitment

Full details about participant recruitment processes have been previously published^(23)^. Low-wage workers were defined as those likely to be affected by the minimum wage in Minneapolis and comparable workers in Raleigh. Participants were eligible if they were (1) 18 years old or older, (2) worked at least 10 h a week at a wage of less than or equal to $11·50/h in Minneapolis/Raleigh OR were employed at that wage within the last six months and were currently seeking work in Minneapolis/Raleigh, (3) planned to serve in the workforce for at least 5 years, (4) agreed to be contacted for follow-up and (5) spoke English or Spanish. Participants were excluded if they were federal/state workers, full-time students or planned to retire or move more than 100 miles away. Wage eligibility was capped at $11·50 an hour or less to include workers earning up to 15 % above the minimum wage at baseline, to include those just above minimum wage who might be affected by a re-scaling of wages^(24)^. We also included those who were not employed at baseline but had been recently employed at low-wage jobs, to account for high turnover and job insecurity in low-wage worker sectors^(25)^. Participants received up to $70 per time point for the completion of all measures.

### Measures

BMI was calculated as weight in kilograms/(height in meters)^2^. Height and weight were collected anthropometrically at T1 and T2. Trained and certified research staff took measures in duplicate on a portable digital scale (Seca model) and portable Schorr stadiometer (Schorr Production, Olney, MD). At T3, weight was collected via scales mailed to participants with the most recent height data used for each participant to calculate BMI.

#### Wages and employment data

Paystubs or other employer documentation was requested at each annual appointment for all current jobs. A data collector verified with participants the employer name, address, start date, job titles, weekly hours worked during the past 2 weeks and hourly wage. Employment status (employed or not employed) was designated based on whether participants were working for pay. Job sector was coded according to the Bureau of Labor Statistics’ guide to Standard Occupational Codes (SOC) for job descriptions, and the North American Industry Classification System for employer sector. For participants who did not provide wage verification, employment information was self-reported. Participants who were currently not employed could submit a paystub from the 6 months prior to their appointment, if they had worked during that period.

#### Survey measures

The online survey was designed to be completed in approximately 25 min. Participants could be assisted by study staff in completing the survey in person (at T1 and T2) or over the phone (at T3). The survey assessed demographics including age, gender, race/ethnicity, education and household size. It also assessed participation in SNAP in the last 30 d (yes/no). Food insecurity was measured by the six-item Household Food Security Survey Module^(26)^ with items summed and classified into food secure (0–1 total score) or food insecure (2–6 total score) categories.

The survey included an abbreviated twenty-two-item Dietary Screener Questionnaire (DSQ)^(27)^. DSQ frequency data was used to estimate daily frequency of intake of fruits and vegetables, whole grain-rich foods (in which the first ingredient is a whole grain), and foods high in added sugars (> 5 g of sugar per serving)^(28,29)^. Participants’ responses to foods that contributed to each of the three food categories were converted into daily frequencies for each food. For example, if a participant reported consuming popcorn ‘2-3 times last month,’ 2·5 (frequency/month) was divided by 30 (days) and assigned a daily frequency value of 0·083, which contributed to the whole grain-rich foods variable. Three variables for each participant were created for the sum of the daily frequencies for all fruit and vegetable foods, all whole grain-rich foods and all foods high in added sugars.

### Analysis

A DiD design was used to detect statistically significant changes in key measures among Minneapolis participants compared with Raleigh participants during the period from T1 in 2018 to T3 in 2020. Each individual who completed an appointment at T1, T2 and T3 was included in the current analysis regardless of their ultimate employment status or their actual wage. To examine missing data, we used Chi-square and *t* tests to test for differences in key demographic characteristics of respondents (defined as those who completed appointments at all three time points) *v*. the full baseline sample of participants in each city. We tested for differences in age, hourly wage, weekly hours worked, household size, pregnancy status, education, race/ethnicity, sex, household income category, working more than one job and job sector according to assigned SOC codes. Next, we examined potential item-non-response bias for wages and BMI. We compared key demographic characteristics among respondents who reported wages at all three time points and those with wage-item non-response in each city; then we compared key demographic characteristics among respondents who reported weight at all three time points and those with weight-item non-response in each city.

To account for potential non-linear changes between the time points, the analysis models and conducts tests of statistical significance for: 1) DiD change from T1 to T2, 2) DiD change from T2 to T3 and 3) joint hypothesis DiD from T1 to T3. An alpha level of 0·05 was used to determine statistical significance.

First, we examined DiD change in three job-related variables (wage, employment status and hours work). Next, we examined DiD change in our primary and secondary outcomes, including BMI, food insecurity, SNAP participation and three dietary variables (servings of fruits and vegetables, servings of whole grain-rich foods and servings of foods high in added sugars). We present unadjusted models as well as models adjusted for potential confounders. Model adjustment included baseline non-time-varying factors (age, sex, race/ethnicity and education) and time-varying factors (number of jobs worked, employment sector, pregnancy status, household size and month of participation). For all outcomes 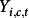 , we used a linear mixed-effects regression model of the form:

Equation 1

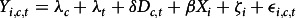




where 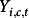 is the outcome of interest, 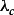 is the city effect (Minneapolis *v*. Raleigh), 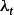 is the time effect in years, 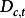 is the city-by-year interaction, 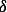 is the intervention effect, 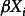 captures the effects of adjustment covariates, 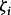 is the participant random effect and 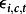 is the residual. The random effect for participant was included to account for correlation between repeated measurements of participants and captures time invariant characteristics of participants. We considered that relevant area-level factors such as Cost of Living Index or area SNAP enrollment could potentially lie on the causal pathway between a minimum wage policy and our measured outcomes; as potential mediators, they were not included in our models.

### Analytic sample

The analytic sample for the analysis included respondents who participated in all three time points (*n* 268 participants in Minneapolis and *n* 312 in Raleigh). Compared with the full sample (*n* 495 in Minneapolis and 479 in Raleigh), respondents were more likely to be female in Raleigh (online Supplementary Table 1). No other differences between respondents and the full sample were detected. The item non-response analysis demonstrated that respondents in Minneapolis who did not provide wage data at one or more time points were more likely to be older compared with those who provided wage data at all three time points; the job sector distribution among those who did not provide wage data was different than for those who provided wage data at all three time points. No other demographic differences in the weight non-responders compared with those who provided weight data at all three time points (online Supplementary Tables 2 and 3).

Baseline demographic and wage data on participants in the analytic sample are presented in Table 1. Participants were, on average, 46·1 years old in Minneapolis and 38·4 in Raleigh, with an average household size of 2·4 in Minneapolis and 2·8 in Raleigh. Approximately half had a high school degree or less in both cities. Non-Hispanic Black participants comprised 63·1 % of the sample in Minneapolis and 80·8 % in Raleigh, white participants comprised 25·1 % in Minneapolis, 11·2 % in Raleigh and a smaller percentage identified as Hispanic, Asian, American Indian/Alaska Native, two or more races or other race in both cities. The Minneapolis sample comprised 53·4 % females *v*. 72·1 % in Raleigh. A large proportion of the sample reported an annual household income ≤ $20 000 (81 % in Minneapolis, 66·7 % in Raleigh). The average hourly wage among workers enrolled in the study at baseline was $10·50 in Minneapolis and $9·50 in Raleigh. In Minneapolis, 11·4 % of participants worked more than one job compared with 10·9 % in Raleigh. At baseline, the most common job types represented were Building and Grounds Cleaning & Maintenance (15·2 %) in Minneapolis and Office and Administrative Support in Raleigh (26·6 %).

**Table 1:** Baseline (T1) demographics and wages (US$) in the analytical sample from WAGE$ study

	Minneapolis (*n* 268)			Raleigh (*n* 312)		
	*n* *	Mean	sd	*n*	Mean	sd
Age	268	46·1	13·7	312	38·4	12·8
Household size	264	2·4	1·8	309	2·8	1·5
Hourly wage (verified or self-reported)	264	10·5	1·3	310	9·5	1·7
Weekly hours worked	259	25·4	10·2	300	33·2	9·5

	*n*	%	*n*	%
Pregnancy status	254		299	
Yes	1	0·4	12	4·0
No	253	99·6	287	96·0
Education	265		311	
Less than high school	58	21·9	28	9·0
High school completed	74	27·9	133	42·8
Some college	37	14·0	30	9·7
Associate/Technical degree	66	24·9	88	28·3
Bachelor’s degree or higher	30	11·3	32	10·3
Race/Ethnicity	263		312	
Hispanic	10	3·8	17	5·5
Non-Hispanic White	66	25·1	35	11·2
Non-Hispanic Black	166	63·1	252	80·8
Non-Hispanic Asian	2	0·8	2	0·6
Non-Hispanic Other	19	7·2	6	1·9
Gender	262		312	
Male	119	45·4	86	27·6
Female	140	53·4	225	72·1
Non-binary	3	1·2	1	0·3
Household income	263		312	
< $5000	66	25·1	54	17·3
$5001–$10 000	74	28·1	64	20·5
$10 001–$20 000	73	27·8	90	28·9
$20 001–$30 000	33	12·6	64	20·5
$30 001–$40 000	7	2·7	25	8·0
$40 001–$50 000	7	2·7	7	2·2
More than $50 001	3	1·1	8	2·6
Working more than one job	268		312	
Yes	31	11·4	34	10·9
No	237	88·4	278	89·1
Job sector	263		305	
Food preparation and serving related	35	13·3	57	18·7
Office and administrative support	24	9·1	81	26·6
Transportation and material moving	34	12·9	25	8·2
Building and grounds cleaning and maintenance	40	15·2	20	6·6
Sales and related occupations	22	8·4	25	8·2
Healthcare support	19	7·2	29	9·5
Protective service	4	1·5	9	3·0
Other	85	32·3	59	19·3

* *n* for non-missing responses.

## Results

**Fig. 1:**
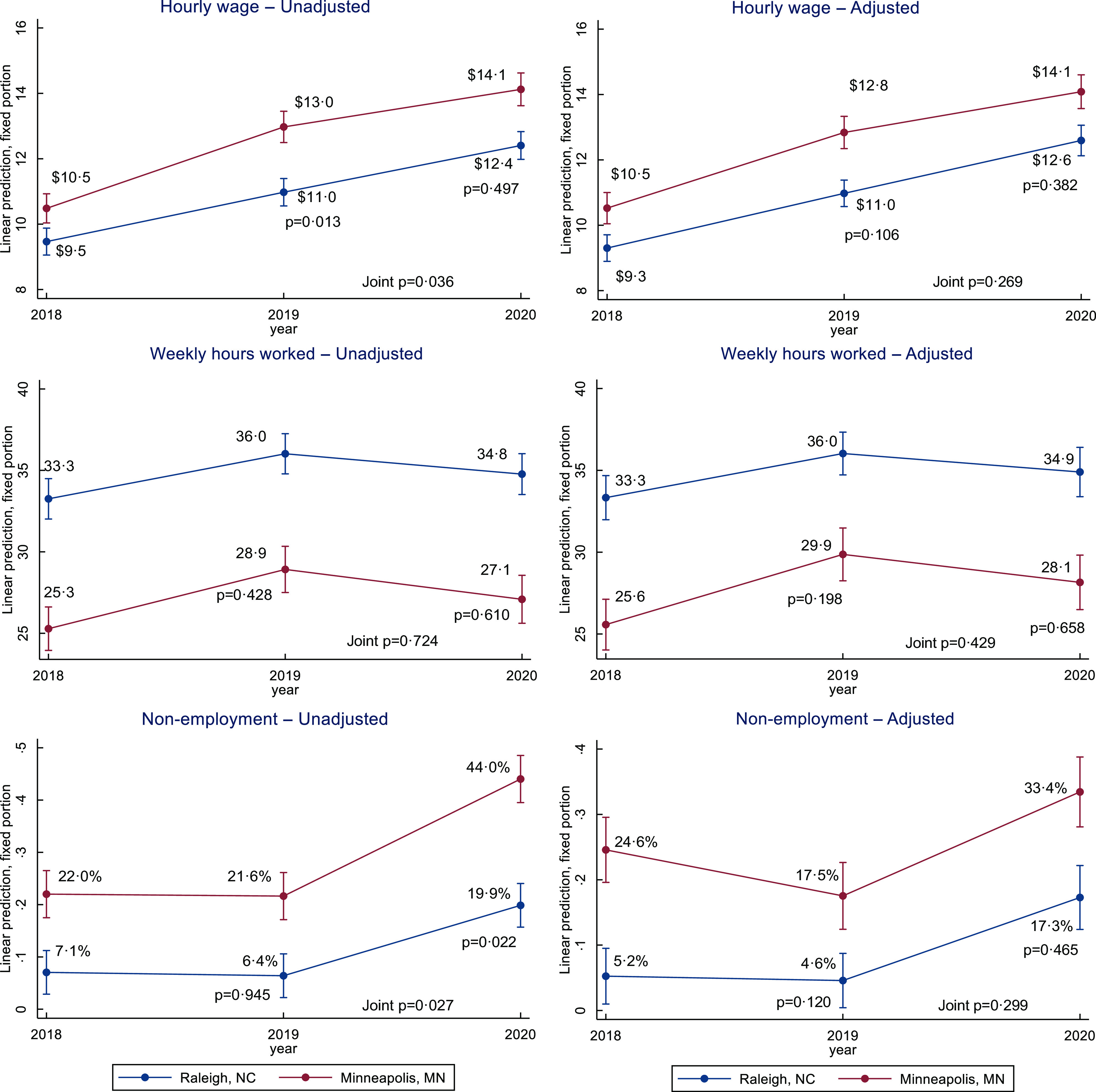
Employment-related changes in the WAGE$ sample, 2018–2020. Trends in hourly wage, weekly hours worked and employment estimated in a difference-in-differences analysis by city. Adjusted models included baseline non-time-varying factors (age, sex, race/ethnicity and education) and time-varying factors (number of jobs worked, employment sector, pregnancy status, household size and month of participation)

**Fig. 2:**
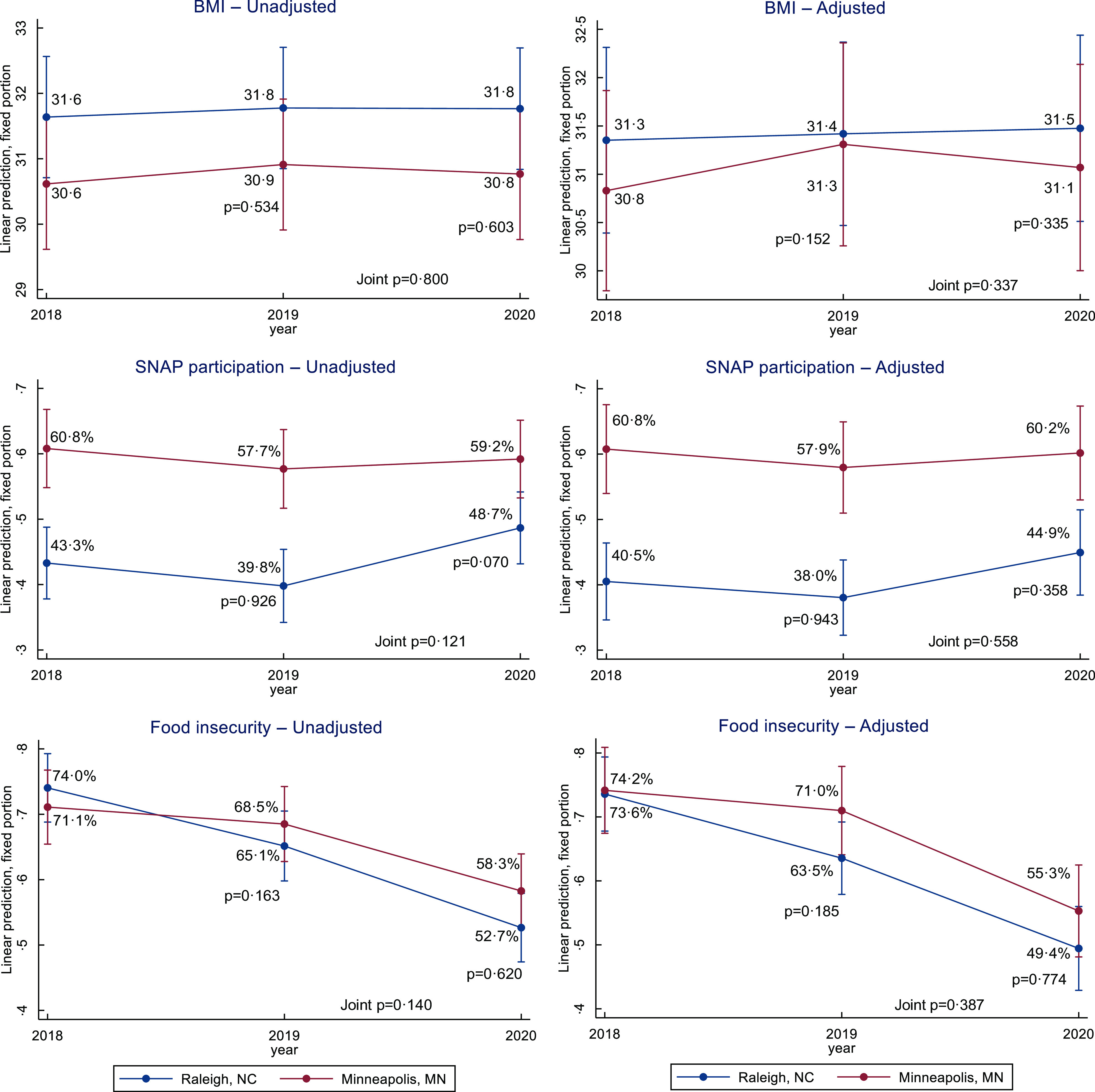
Weight and food insecurity changes in the WAGE$ sample, 2018–2020. Trends of BMI, Supplemental Nutrition Assistance Program (SNAP) participation and food insecurity estimated in a difference-in-difference analysis by city. Adjusted models included baseline non-time-varying factors (age, sex, race/ethnicity and education) and time-varying factors (number of jobs worked, employment sector, pregnancy status, household size and month of participation)

**Fig. 3:**
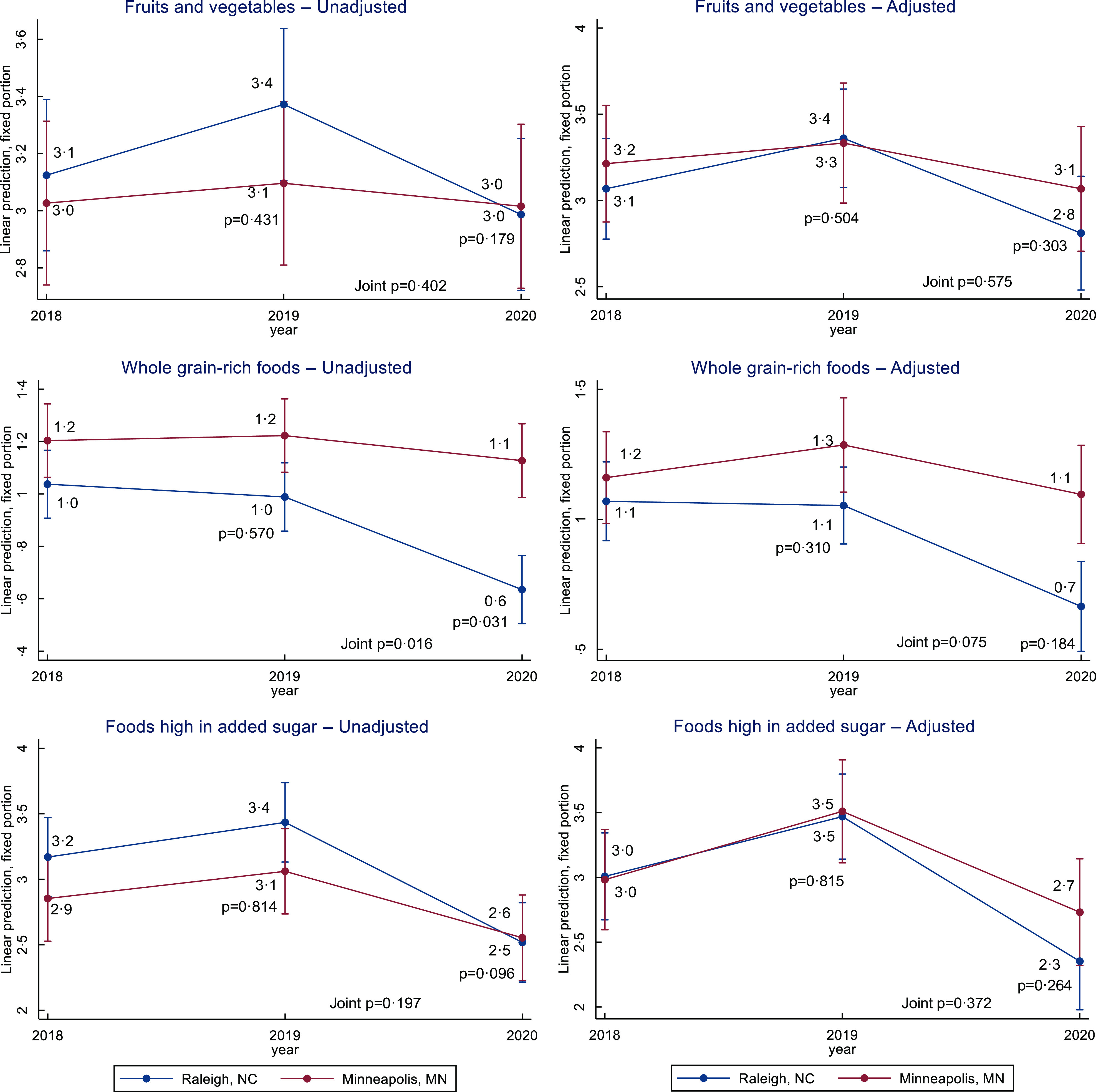
Diet quality changes in the WAGE$ sample, 2018–2020. Trends in daily intake of fruits and vegetables, of whole grain-rich foods, and of foods high in added sugars, in servings per day estimated in a difference-in-difference analysis by city. Adjusted models included baseline non-time-varying factors (age, sex, race/ethnicity and education) and time-varying factors (number of jobs worked, employment sector, pregnancy status, household size and month of participation)

Results of the DiD analysis of employment variables and primary and secondary outcomes are presented in Table 2. Trends are presented in Figs 1–3.

**Table 2:** Description of employment variables and primary and secondary outcomes and city-specific changes from T1 to T2, from T2 and T3 and jointly from T1 to T3 in the WAGE$ sample

	Minneapolis (*n* 268)												Raleigh (*n* 312)											
	T1			T2			T3			T1 to T2	T2 to T3	Joint	T1			T2			T3			T1 to T2	T2 to T3	Joint
	*n* *	Mean	sd	*n*	Mean	sd	*n*	Mean	sd	*P* value for change			*n*	Mean	sd	*n*	Mean	sd	*n*	Mean	sd	*P*-value for change		
Hourly wage	264	10·5	1·3	226	13·0	4·9	205	14·1	4·5	< 0·001	< 0·001	< 0·001	310	9·5	1·7	298	11·0	3·5	290	12·4	5·0	< 0·001	< 0·001	< 0·001
Weekly hours worked	259	25·4	10·2	225	29·1	13·2	207	27·1	12·8	< 0·001	0·036	< 0·001	300	33·2	9·5	303	36·1	10·0	291	34·8	10·7	< 0·001	0·090	< 0·001
BMI	268	30·6	8·0	267	30·9	8·2	253	30·8	8·8	0·110	0·445	0·223	312	31·6	8·5	311	31·8	8·4	302	31·8	8·4	0·418	0·950	0·698
Diet quality^†^																								
Fruits and vegetables	266	3·0	2·3	267	3·1	2·6	265	3·0	2·2	0·676	0·629	0·867	312	3·1	2·4	308	3·4	2·7	309	3·0	2·0	0·108	0·013	0·045
Whole grain-rich foods	266	1·2	1·2	267	1·2	1·3	265	1·1	1·0	0·827	0·277	0·552	312	1·0	1·4	308	1·0	1·2	309	0·6	0·8	0·548	< 0·001	< 0·001
Foods high in added sugar	266	2·9	2·5	267	3·1	3·0	265	2·6	2·5	0·248	0·005	0·015	312	3·2	2·8	308	3·4	3·2	309	2·5	2·2	0·111	< 0·001	< 0·001
	*n*	%		*n*	%		*n*	%					*n*	%		*n*	%		*n*	%				
Employed	268			268			268			0·896	< 0·001	< 0·001	312			312			312			0·809	< 0·001	< 0·001
Yes	209	78·0		210	78·4		150	56·0					290	93·0		292	93·6		250	80·1				
No	59	22·0		58	21·6		118	44·0					22	7·1		20	6·4		62	19·9				
SNAP^‡^ participation	259			253			264			0·301	0·614	0·578	311			294			309			0·206	0·001	0·006
Yes	159	61·4		147	58·1		156	59·1					134	43·1		118	40·1		151	48·9				
No	98	37·8		106	41·9		107	40·5					175	56·3		174	59·2		157	50·8				
Not sure	2	0·8					1	0·4					2	0·6		2	0·7		1	0·3				
Food insecurity	266			258			265			0·434	0·002	< 0·001	312			296			310			0·004	< 0·001	< 0·001
Yes	189	71·1		177	68·6		155	58·5					231	74·0		193	65·2		163	52·6				
No	77	29·0		81	31·4		110	41·5					81	26·0		103	34·8		147	47·4				

* *n* for non-missing responses.

† Daily frequency of intake (servings/day).

‡ Supplemental Nutrition Assistance Program.

### Changes in employment-related variables

Between T1 and T3, mean hourly wage increased in Minneapolis from $10·50 in T1 to $14·10 at T3 (*P* < 0·001) In Raleigh, it increased from $9·50 to $12·40 (*P* < 0·001). The DiD analysis of wages indicated that the average wage increased to a greater degree in Minneapolis compared with Raleigh in the unadjusted model (*P* = 0·036), but not in the adjusted model (*P* = 0·269). Unadjusted models showed a greater increase in wages in Minneapolis between T1 and T2 (*P* = 0·013), but not between T2 and T3 (*P* = 0·497).

Changes in the number of hours worked per week were non-linear. The mean number of hours worked increased in Minneapolis from 25·4 at T1 to 29·1 at T2 (*P* < 0·001) and then decreased to 27·1 at T3 (*P* = 0·03). In Raleigh, the number of hours worked increased from 33·2 at T1 to 36·1 at T2 (*P* < 0·001) and then decreased non-significantly to 34·8 at T3 (*P* = 0·090). The DiD in number of hours worked was not statistically significant in any model or at any time point.

In Minneapolis, non-employment did not change between T1 (22 %) and T2 (21·6 %), but more than doubled between T2 and T3 to 44 % (*P* < 0·001). In Raleigh, non-employment was also relatively stable between T1 (7·1 %) and T2 (6·4 %) and more than doubled to 19·9 % between T2 and T3 (*P* < 0·001). Between T2 and T3, the DiD increase in non-employment was greater in Minneapolis compared with Raleigh in the unadjusted model (*P* = 0·022), but not in the adjusted model (*P* = 0·465).

### Changes in BMI

Average BMI was nearly unchanged in Minneapolis, from 30·6 in T1 to 30·8 in T3 (*P* = 0·223). In the same period, in Raleigh, average BMI was nearly unchanged from 31·6 to 31·8 (*P* = 0·698). The DiD analysis showed no statistically significant differences in changes in any model or at any time point.

### Changes in diet-related variables

SNAP participation in Minneapolis was 61·4 % at T1 and remained relatively stable at T2 (58·1 %, *P* = 0·301) and from T2 to T3 (59·1 %, *P* = 0·614). SNAP participation in Raleigh was 43·1 % at T1, decreased non-significantly to 40·1 % at T2 (*P* = 0·206) and then increased to 48·9 % at T3 (*P* = 0·001). The DiD analysis of SNAP participation was not statistically significant in any model or at any time point.

Food insecurity was 71·1 % in Minneapolis at T1, decreased non-significantly to 68·6 at T2 (*P* = 0·434) and then decreased to 58·5 in T3 (*P* = 0·002). In Raleigh, food insecurity was 74·0 % in T1, decreased to 65·2 in T2 (*P* = 0·004) and then decreased further to 52·6 at T3 (*P* < 0·001). The DiD in food insecurity was not statistically significant in any model or at any time point.

Daily frequency of intake for fruits and vegetables was relatively stable in Minneapolis from T1 to T3 (3·0 servings/day) (*P* = 0·867). In Raleigh, daily frequency of intake for fruits and vegetables was 3·1 servings/day in T1, increased non-significantly to 3·4 servings/day in T2 and then decreased to 3·0 servings/day in T3 (*P* = 0·013). The DiD in daily frequency of intake for fruits and vegetables was not statistically significant in any model or at any time point.

Daily frequency of intake for whole grain-rich foods in Minneapolis did not change from T1 (1·2 servings/day) to T3 (1·1 servings/day) (*P* = 0·552). In Raleigh, daily frequency of intake for whole grain-rich foods was stable from T1 to T2 (1·0 servings/day) (*P* = 0·548) and then decreased to 0·6 servings/day at T3 (*P* < 0·001). Between T2 and T3, the DiD decrease in daily frequency of intake for whole grain-rich foods was smaller in Minneapolis than in Raleigh for the unadjusted model (*P* = 0·031) but not for the adjusted model (*P* = 0·184).

In Minneapolis, daily frequency of intake for foods high in added sugar was 2·9 servings/day at T1, increased non-significantly to 3·1 at T2 (*P* = 0·248) and then decreased to 2·6 servings/day at T3 (*P* = 0·005). In Raleigh, daily frequency of intake for foods high in added sugar also remained unchanged from T1 (3·2 servings/day) to T2 (3·4 servings/day) and then decreased to 2·5 servings/day at T3 (*P* < 0·001). The DiD in daily frequency of intake for foods high in added sugar was not statistically significant in any model or at any time point.

## Discussion

This analysis aimed to measure the causal impacts of an increase in the minimum wage on diet-related measures. The DiD model offers a quasi-experimental comparison of the effects of the increases in the minimum wage in an intervention group (Minneapolis) and a control group (Raleigh). Our central conclusion is that there were no beneficial or adverse effects of increases in the minimum wage on the health outcomes measured during the early phases of policy implementation, which was also a period when major economic and social changes were occurring.

Results did not show statistically significant differences in changes in employment variables, BMI or diet-related outcomes in Minneapolis compared with the control city. However, several within-city trends were noteworthy across the two cities from 2018 to 2020. These trends generally indicate an increase in hourly wage, stable BMI, a decrease in food insecurity and non-linear trends in employment, hours worked, SNAP participation and dietary outcomes. Results were broadly consistent with an earlier analysis of 2018–2019 changes in wages and diet quality^(30)^.

Hourly wages rose in each observation period in both cities, with the average hourly wage increasing $3·60 between 2018 and 2020 in Minneapolis and $2·90 in Raleigh in the same period. Hourly wage increases from 2018 to 2019 were significantly higher in Minneapolis than in Raleigh until the model was adjusted for potential confounders, including job sector. It is possible that workers in Minneapolis changed jobs to sectors with higher average wages during the first observation period. Broadly, trends in hourly earnings observed in the study were similar to those observed throughout the state according to Minnesota and North Carolina BLS data^(31,32)^; moreover, both states’ trends were similar to national trends^(33)^.

It has been suggested that national wage trends in 2020 can be largely accounted for by the disproportionate exit of low-wage workers from the workforce due to the pandemic^(34)^. Indeed, nationally, the highest job losses were by far observed in low-wage employment sectors^(35)^. This could be a consideration even within the WAGE$ sample, as the lowest earning workers may have been the most likely to lose jobs, which could account for a portion of the observed increase in average hourly wage between 2019 and 2020. On the whole, because trends in Raleigh and Minneapolis were similar, it suggests that relatively similar wage increases may have been observed in Minneapolis even in the absence of the city’s Minimum Wage Ordinance.

A recent study by the Federal Reserve Bank of Minneapolis^(36)^ examined the economic effects of the Minneapolis Minimum Wage Ordinance using synthetic DiD estimation; that study demonstrated some sector-specific effects on economic outcomes through the start of 2020. For instance, wages increased by 10·7 % in Administration in Support, while other industries, such as Accommodation and Food Service, saw no wage growth. However, a key difference between the two studies was that the WAGE$ sample was limited to a sample earning close to minimum wage, whereas the Federal Reserve analysis included all but the top 25 % of earners.

A remarkable trend in both cities in the WAGE$ study was the twofold increase in the proportion of participants who reported not being employed in 2020 compared with 2019. Soaring unemployment in 2020 reflected national labour trends^(37)^, resulting from business closures and layoffs during the COVID-19 pandemic. This period was marked not only by the COVID-19 pandemic but also by repercussions from the murder of George Floyd where, as the epicenter of both the murder itself and the civil unrest that followed, Minneapolis saw both temporary and permanent business closures throughout the central corridors of the city^(38)^. This social and economic context in Minneapolis is likely to be relevant in the broader interpretation of other WAGE$ trends as well, as the period following the civil uprising in Minneapolis was characterised by complexities relevant to the health of its residents. As one example, the period saw heightened social action that yielded new emergency food distribution centers^(39)^, perhaps blunting the more severe economic hardship, food insecurity or dietary changes that may have otherwise been expected in this context.

In both cities, rates of food insecurity were consistently higher than expected; at all time points, more than half of the sample reported food insecurity, whereas nationally, food insecurity is approximately 35 % among those below the poverty line^(40)^. These results reinforce the social, financial and health vulnerabilities among low-wage workers that have been described in previous literature^(41–44)^. The observed decline in food insecurity rates by more than 10 percentage points from 2019 to 2020 in both cities is likely related to the federal government economic relief measures due to the COVID-19 pandemic. In March of 2020, as part of the Families First Coronavirus Response Act, the USDA authorised a series of SNAP expansions and flexibilities, which included emergency allotments to increase households’ benefit amounts to the maximum allowed. This change affected both Minnesota and North Carolina^(45)^. For those already receiving SNAP benefits, this change would not have been reflected in SNAP participation rates, but may have been reflected in the decline in rates of food insecurity. In Raleigh, however, the proportion of participants receiving SNAP benefits increased by 9 percentage points in 2020. This change may reflect additional flexibilities in requirements for SNAP eligibility during the pandemic^(46)^ that disproportionately affected North Carolina participants compared with Minnesota participants. Specifically, prior to the Families First Coronavirus Response Act, North Carolina had a state moratorium on waiving SNAP work requirements for able-bodied adults without dependents^(47)^, which served as a barrier for benefit receipt for many low-income Raleigh households^(42)^. The changes in SNAP benefits observed in this two-site study are an example of how COVID-19-era changes to a single program had both universal and state-specific effects.

The federal COVID-19 response was not limited to an expansion of SNAP, but also included other measures to support the economic stability of households, such as additional unemployment benefits and stimulus checks. It is notable that some decline in food insecurity was apparent in the study sample between 2018 and 2019, possibly as a result of wage growth and stable employment; however, the steep decline between 2018 and 2019, along with striking concurrent job losses, suggests that the COVID-19 federal policy response was instrumental in easing the burden of food insecurity during the pandemic. National food insecurity data provide further evidence to support the notion that federal policy supports mitigated an increase in food insecurity in the aggregate US population during the pandemic^(48)^. National trends in food insecurity in 2020 did not, however, suggest a decline in groups who are strongly represented in the WAGE$ sample (i.e. lower income and mostly non-Hispanic Black households), which makes the observed trends in the current study unexpected.

No major changes in BMI were observed during the study period. With the study sample size, statistical power is limited for detecting the small changes in BMI that might be expected to occur over a 2-year period during which wages are gradually rising. Results from a qualitative study of a subset of the WAGE$ sample in 2019 (after the first phase of implementation) also suggest that incremental increases in the minimum wage may not be enough to meaningfully affect household finances or subsequent health outcomes. While many participants were guardedly optimistic that the policy could be somewhat helpful, they also expressed concerns about rising housing costs, made a distinction between $15 and hour wage and a living wage^(42)^.

Other specific diet-related patterns were non-linear and somewhat more difficult to explain. For example, it is not clear why consumption of fruit and vegetables, whole-grain rich foods and foods high in added sugar all decreased in 2020 in one or both cities. However, an array of concerns surrounding food access and shopping behaviours have been identified among U.S. consumers as a result of the COVID-19 pandemic, including concerns around rising food prices, food shortages and challenges using SNAP benefits online^(49)^. These concerns could have resulted in unusual food behaviour or reporting of food consumption in 2020 in both sites, rather than the overall shift from less healthy to more healthy foods in the Minneapolis sample that was expected.

### Limitations

In this two-site study, local policies and social contexts limit the generalisability of our findings. Moreover, the COVID-19 pandemic brought broad disruption in pre-pandemic trends to numerous measures collected in the study. Social and contextual differences between the cities are more difficult to measure quantitatively and must be factored into the interpretation of the results through planned qualitative data collection and mixed-methods analysis. However, a previous analysis of baseline WAGE$ data found that baseline differences in BMI between Black women in the two cities were almost entirely explained by demographic differences – namely differences in age and education – rather than by contextual differences^(50)^. Indeed, baseline differences in Minneapolis and Raleigh samples have been noted in previous publications^(20,50)^ and are likely attributable to unavoidable differences in the implementation of community recruitment strategies in the two cities^(23)^. The analysis was also not powered to look at sector-specific effects. Finally, while we adjusted for known demographic differences between the site, and for the most part, adjustment for demographic factors did not change the results, residual confounding by other unmeasured factors that affect behaviours is possible.

### Conclusions

Findings from this mid-point evaluation provide no evidence of beneficial or adverse effects of the Minimum Wage Ordinance on diet-related variables among low-wage workers. The study evaluated the early effects of one city’s minimum wage policy during a period of major economic and social change; as such, results cannot be generalised to all income interventions, or to local minimum wage ordinances implemented in a different milieu or with a different wage level.

While there were no notable between-city differences, across cities there was an observed increase in hourly wage, stable BMI, an overall decrease in food insecurity and non-linear trends in employment, hours worked, SNAP participation and dietary outcomes. The COVID-19 pandemic and other unanticipated events of 2020, including far-reaching federal relief economic measures and the murder of George Floyd in Minneapolis, likely contributed to the observed trends. Additional analyses are underway to more formally compare COVID-19 policy implementation in the two cities, as well as the impact of relief measure receipt on outcomes like food insecurity in this sample of low-wage workers.

## Supplementary material

For supplementary material accompanying this paper visit https://doi.org/10.1017/S1368980023001520. click here to view supplementary material
