# Supplementary material for: Early results of a natural experiment evaluating the effects of a local minimum wage policy on the diet-related health of low-wage workers, 2018–2020
Source: Public Health Nutr. 2023 Aug 7;26(11):2573–85. doi: 10.1017/S1368980023001520 (PMC10641626; doi:10.1017/S1368980023001520)
Supplement: Supplementary file 1 [file S1368980023001520sup001.docx]

**Supplemental Table 1.** Comparison of baseline demographic characteristics of participants in the analytical sample (respondents at all three annual appointments) and the full sample (all enrolled participants)

|  | **Minneapolis (n=495)** | | | **Raleigh (n=479)** | | |
| --- | --- | --- | --- | --- | --- | --- |
|  | **N^a^** | **Mean (SD)** | **p-value** | **N** | **Mean (SD)** | **p-value** |
| Age | 494 | 45.0 (13.7) | 0.303 | 479 | 37.8 (12.8) | 0.520 |
| Household size | 488 | 2.4 (1.7) | 0.851 | 470 | 2.8 (1.6) | 0.689 |
| Hourly wage | 484 | 10.4 (1.5) | 0.492 | 471 | 9.3 (1.7) | 0.253 |
| Weekly hours worked | 472 | 26.6 (10.4) | 0.145 | 462 | 32.9 (9.4) | 0.597 |
|  | **N** | **%** |  | **N** | **%** |  |
| Pregnancy status | 474 |  | 0.653 | 460 |  | 0.702 |
| Yes | 1 | 0.2 |  | 16 | 3.5 |  |
| No | 473 | 99.8 |  | 444 | 96.5 |  |
| Education | 491 |  | 0.587 | 475 |  | 0.458 |
| Less than high school | 103 | 21.0 |  | 62 | 13.1 |  |
| High school completed | 164 | 33.4 |  | 206 | 43.4 |  |
| Some college | 62 | 12.6 |  | 40 | 8.4 |  |
| Associate/Technical degree | 117 | 23.8 |  | 122 | 25.7 |  |
| Bachelor’s degree or higher | 45 | 9.2 |  | 45 | 9.5 |  |
| Race/Ethnicity | 487 |  | 0.554 | 479 |  | 0.988 |
| Hispanic | 26 | 5.3 |  | 26 | 5.4 |  |
| Non-Hispanic White | 102 | 20.9 |  | 58 | 12.1 |  |
| Non-Hispanic Black | 315 | 64.7 |  | 384 | 80.2 |  |
| Non-Hispanic Asian | 2 | 0.4 |  | 2 | 0.4 |  |
| Non-Hispanic Other | 42 | 8.6 |  | 9 | 1.9 |  |
| Gender | 487 |  | 0.247 | 479 |  | 0.041 |
| Male | 250 | 51.3 |  | 173 | 36.1 |  |
| Female | 234 | 48.1 |  | 304 | 63.5 |  |
| Non-binary | 3 | 0.6 |  | 2 | 0.4 |  |
| Household income | 486 |  | 0.843 | 474 |  | 0.873 |
| Less than $5,000 | 146 | 30.0 |  | 98 | 20.7 |  |
| $5,001 to $10,000 | 132 | 27.2 |  | 100 | 21.1 |  |
| $10,001 to $20,000 | 127 | 26.1 |  | 139 | 29.3 |  |
| $20,001 to $30,000 | 49 | 10.1 |  | 86 | 18.1 |  |
| $30,001 to $40,000 | 12 | 2.5 |  | 31 | 6.5 |  |
| $40,001 to $50,000 | 14 | 2.9 |  | 10 | 2.1 |  |
| More than $50,001 | 6 | 1.2 |  | 10 | 2.1 |  |
| Working more than one job | 495 |  | 0.590 | 479 |  | 0.555 |
| Yes | 51 | 10.3 |  | 46 | 9.6 |  |
| No | 444 | 89.7 |  | 433 | 90.4 |  |
| Job sector | 482 |  | 0.820 | 467 |  | 0.542 |
| Food Preparation & Serving Related | 75 | 15.6 |  | 97 | 20.8 |  |
| Office and Administrative Support | 33 | 6.9 |  | 104 | 22.3 |  |
| Transportation and Material Moving | 78 | 16.2 |  | 59 | 12.6 |  |
| Building and Grounds Cleaning & Maintenance | 72 | 14.9 |  | 26 | 5.6 |  |
| Sales & Related Occupations | 38 | 7.9 |  | 40 | 8.6 |  |
| Healthcare Support | 29 | 6.0 |  | 39 | 8.4 |  |
| Protective Service | 9 | 1.9 |  | 16 | 3.4 |  |
| Other | 148 | 30.7 |  | 86 | 18.4 |  |

**Supplemental Table 2.** Hourly wage-item non-response analysis, comparing key demographic characteristics among respondents who reported wages at all three time points and those with wage-item non-response in each city of the WAGE$ analytical sample

|  | Minneapolis (n=268) | | | | | Raleigh (n=312) | | | | |
| --- | --- | --- | --- | --- | --- | --- | --- | --- | --- | --- |
|  | Reported | | Non-response | |  | Reported | | Non-response | |  |
|  | n=188 | | n=80 | |  | n=275 | | n=37 | |  |
| Variables | N | Mean (SD) | N | Mean (SD) | p-value | N | Mean (SD) | N | Mean (SD) | p-value |
| Age | 188 | 44.7 (14.0) | 80 | 49.3 (12.5) | 0.012 | 275 | 37.9 (12.6) | 37 | 42.1 (13.4) | 0.059 |
| Household size | 185 | 2.4 (1.8) | 79 | 2.2 (1.5) | 0.452 | 272 | 2.8 (1.5) | 37 | 2.7 (1.6) | 0.741 |
| Hourly wage | 188 | 10.5 (1.3) | 76 | 10.3 (1.2) | 0.290 | 275 | 9.5 (1.8) | 35 | 9.3 (1.3) | 0.657 |
| Weekly hours worked | 183 | 25.9 (10.6) | 76 | 24.3 (9.1) | 0.253 | 264 | 33.4 (9.0) | 36 | 32.1 (12.9) | 0.432 |
| Body Mass Index (BMI) | 188 | 30.6 (7.9) | 80 | 30.7 (8.4) | 0.964 | 275 | 31.5 (8.6) | 37 | 32.4 (7.2) | 0.557 |
|  | N | % | N | % |  | N | % | N | % |  |
| Education | 186 |  | 79 |  | 0.138 | 274 |  | 37 |  | 0.276 |
| Less than high school | 45 | 19.4 | 22 | 27.9 |  | 22 | 8.0 | 6 | 16.2 |  |
| High school completed | 49 | 26.3 | 25 | 31.7 |  | 119 | 43.4 | 14 | 37.8 |  |
| Some college | 30 | 16.1 | 7 | 8.9 |  | 28 | 10.2 | 2 | 5.4 |  |
| Associate/Technical degree | 46 | 24.7 | 20 | 25.3 |  | 75 | 27.4 | 13 | 35.1 |  |
| Bachelor’s degree or higher | 25 | 13.4 | 5 | 6.3 |  | 30 | 11.0 | 2 | 5.4 |  |
| Race/Ethnicity | 184 |  | 79 |  | 0.345 | 275 |  | 37 |  | 0.262 |
| Hispanic | 7 | 3.8 | 3.8 | 3.8 |  | 17 | 6.2 | 0 | 0.0 |  |
| Non-Hispanic White | 52 | 28.3 | 14 | 17.7 |  | 31 | 11.3 | 4 | 10.8 |  |
| Non-Hispanic Black | 111 | 60.3 | 55 | 69.6 |  | 221 | 80.4 | 31 | 83.8 |  |
| Non-Hispanic Asian | 2 | 1.1 | 0 | 0.0 |  | 2 | 0.7 | 0 | 0.0 |  |
| Non-Hispanic Other | 12 | 6.5 | 7 | 8.9 |  | 4 | 1.5 | 2 | 5.4 |  |
| Gender | 182 |  | 79 |  | 0.331 | 275 |  | 37 |  | 0.419 |
| Male | 81 | 44.5 | 38 | 47.5 |  | 79 | 28.7 | 7 | 18.9 |  |
| Female | 100 | 55.0 | 40 | 50.0 |  | 195 | 70.9 | 30 | 81.1 |  |
| Non-binary | 1 | 0.6 | 2 | 2.5 |  | 1 | 0.4 | 0 | 0.0 |  |
| Household income | 185 |  | 78 |  | 0.387 | 275 |  | 37 |  | 0.646 |
| Less than $5,000 | 48 | 26.0 | 18 | 23.1 |  | 47 | 17.1 | 7 | 18.9 |  |
| $5,001 to $10,000 | 55 | 29.7 | 19 | 24.4 |  | 54 | 19.6 | 10 | 27.0 |  |
| $10,001 to $20,000 | 47 | 25.4 | 26 | 33.3 |  | 79 | 28.7 | 11 | 29.7 |  |
| $20,001 to $30,000 | 20 | 10.8 | 13 | 16.7 |  | 60 | 21.8 | 4 | 10.8 |  |
| $30,001 to $40,000 | 6 | 3.2 | 1 | 1.3 |  | 21 | 7.6 | 4 | 10.8 |  |
| $40,001 to $50,000 | 6 | 3.2 | 1 | 1.3 |  | 7 | 2.6 | 0 | 0.0 |  |
| More than $50,001 | 3 | 1.6 | 0 | 0.0 |  | 7 | 2.6 | 1 | 2.7 |  |
| Working more than one job | 188 |  | 80 |  | 0.174 | 275 |  | 37 |  | 0.253 |
| Yes | 25 | 13.3 | 6 | 7.5 |  | 32 | 11.6 | 2 | 5.4 |  |
| No | 163 | 86.7 | 74 | 92.5 |  | 243 | 88.4 | 35 | 94.6 |  |
| Job sector | 185 |  | 78 |  | 0.006 | 270 |  | 35 |  | 0.554 |
| Food Preparation & Serving Related | 26 | 14.1 | 9 | 11.5 |  | 50 | 18.5 | 7 | 20.0 |  |
| Office and Administrative Support | 20 | 10.8 | 4 | 5.1 |  | 73 | 27.0 | 8 | 22.9 |  |
| Transportation and Material Moving | 24 | 13.0 | 10 | 12.8 |  | 25 | 9.3 | 0 | 0.0 |  |
| Building and Grounds Cleaning & Maintenance | 18 | 9.7 | 22 | 28.2 |  | 16 | 5.9 | 4 | 11.4 |  |
| Sales & Related Occupations | 19 | 10.3 | 3 | 3.9 |  | 22 | 8.2 | 3 | 8.6 |  |
| Healthcare Support | 16 | 8.7 | 3 | 3.9 |  | 24 | 8.9 | 5 | 14.3 |  |
| Protective Service | 2 | 1.1 | 2 | 2.6 |  | 8 | 3.0 | 1 | 2.9 |  |
| Other | 60 | 32.4 | 25 | 32.1 |  | 52 | 19.3 | 7 | 20.0 |  |

**Supplemental Table 3.** Weight-item non-response analysis, comparing key demographic characteristics among respondents who reported weight at all three time points and those with weight-item non-response in each city of the WAGE$ analytical sample

|  | Minneapolis (MN) n=268 | | | | | Raleigh (NC) n=312 | | | | |
| --- | --- | --- | --- | --- | --- | --- | --- | --- | --- | --- |
|  | Reported | | Non-response | |  | Reported | | Non-response | |  |
|  | n=252 | | n=16 | |  | n=301 | | n=11 | |  |
| Variables | N | Mean (SD) | N | Mean (SD) | p-value | N | Mean (SD) | N | Mean (SD) | p-value |
| Age | 252 | 46.1 (13.7) | 16 | 45.1 (13.8) | 0.757 | 301 | 38.3 (12.8) | 11 | 39.7 (13.9) | 0.719 |
| Household size | 248 | 2.4 (1.8) | 16 | 1.9 (1.5) | 0.329 | 298 | 2.8 (1.5) | 11 | 2.3 (1.3) | 0.258 |
| Hourly wage | 248 | 10.5 (1.3) | 16 | 10.5 (0.7) | 0.835 | 299 | 9.5 (1.7) | 11 | 9.0 (1.3) | 0.385 |
| Weekly hours worked | 243 | 25.6 (10.1) | 16 | 22.9 (11.3) | 0.314 | 290 | 33.3 (9.6) | 10 | 31.5 (7.6) | 0.561 |
| Body Mass Index (BMI) | 252 | 30.6 (8.1) | 16 | 31.5 (6.1) | 0.649 | 301 | 31.7 (8.5) | 11 | 39.7 (13.9) | 0.468 |
|  | N | % | N | % |  | N | % | N | % |  |
| Education | 249 |  | 16 |  | 0.368 | 300 |  | 11 |  | 0.397 |
| Less than high school | 56 | 22.5 | 2 | 12.5 |  | 26 | 8.7 | 2 | 18.2 |  |
| High school completed | 71 | 28.5 | 3 | 18.8 |  | 130 | 43.3 | 3 | 27.3 |  |
| Some college | 34 | 13.7 | 3 | 18.8 |  | 29 | 9.7 | 1 | 9.1 |  |
| Associate/Technical degree | 62 | 24.9 | 4 | 25.0 |  | 83 | 27.7 | 5 | 45.5 |  |
| Bachelor’s degree or higher | 26 | 10.4 | 4 | 25.0 |  | 32 | 10.7 | 0 | 0.0 |  |
| Race/Ethnicity | 248 |  | 15 |  | 0.723 | 301 |  | 11 |  | 0.607 |
| Hispanic | 9 | 3.6 | 1 | 6.7 |  | 17 | 5.7 | 0 | 0.0 |  |
| Non-Hispanic White | 61 | 24.6 | 5 | 33.3 |  | 35 | 11.6 | 0 | 0.0 |  |
| Non-Hispanic Black | 157 | 63.3 | 9 | 60.0 |  | 241 | 80.1 | 11 | 100.0 |  |
| Non-Hispanic Asian | 2 | 0.8 | 0 | 0.0 |  | 2 | 0.7 | 0 | 0.0 |  |
| Non-Hispanic Other | 19 | 7.7 | 0 | 0.0 |  | 6 | 2.0 | 0 | 0.0 |  |
| Gender | 247 |  | 15 |  | 0.764 | 301 |  | 11 |  | 0.790 |
| Male | 111 | 44.9 | 8 | 53.3 |  | 82 | 27.2 | 4 | 36.4 |  |
| Female | 133 | 53.9 | 7 | 46.7 |  | 218 | 72.4 | 7 | 63.6 |  |
| Non-binary | 3 | 1.2 | 0 | 0.0 |  | 1 | 0.3 | 0 | 0.0 |  |
| Household income | 247 |  | 16 |  | 0.204 | 301 |  | 11 |  | 0.678 |
| Less than $5,000 | 61 | 24.7 | 5 | 31.3 |  | 51 | 16.9 | 3 | 27.3 |  |
| $5,001 to $10,000 | 71 | 28.7 | 3 | 18.8 |  | 62 | 20.6 | 2 | 18.2 |  |
| $10,001 to $20,000 | 70 | 28.3 | 3 | 18.8 |  | 88 | 29.2 | 2 | 18.2 |  |
| $20,001 to $30,000 | 31 | 12.6 | 2 | 12.5 |  | 60 | 19.9 | 4 | 36.4 |  |
| $30,001 to $40,000 | 5 | 2.0 | 2 | 12.5 |  | 25 | 8.3 | 0 | 0.0 |  |
| $40,001 to $50,000 | 6 | 2.4 | 1 | 6.3 |  | 7 | 2.3 | 0 | 0.0 |  |
| More than $50,001 | 3 | 1.2 | 0 | 0.0 |  | 8 | 2.7 | 0 | 0.0 |  |
| Working more than one job | 252 |  | 16 |  | 0.083 | 301 |  | 11 |  | 0.076 |
| Yes | 27 | 10.7 | 4 | 25.0 |  | 31 | 10.3 | 3 | 27.3 |  |
| No | 225 | 89.3 | 12 | 75.0 |  | 270 | 89.7 | 8 | 72.7 |  |
| Job sector |  |  | 16 |  | 0.827 | 294 |  |  |  | 0.926 |
| Food Preparation & Serving Related | 32 | 13.0 | 3 | 18.8 |  | 54 | 18.4 | 3 | 27.3 |  |
| Office and Administrative Support | 23 | 9.3 | 1 | 6.3 |  | 79 | 26.9 | 2 | 18.2 |  |
| Transportation and Material Moving | 32 | 13.0 | 2 | 12.5 |  | 25 | 8.5 |  |  |  |
| Building and Grounds Cleaning & Maintenance | 37 | 15.0 | 3 | 18.8 |  | 19 | 6.5 | 1 | 9.1 |  |
| Sales & Related Occupations | 21 | 8.5 | 1 | 6.3 |  | 24 | 8.2 | 1 | 9.1 |  |
| Healthcare Support | 18 | 7.3 | 1 | 6.3 |  | 28 | 9.5 | 1 | 9.1 |  |
| Protective Service | 3 | 1.2 | 1 | 6.3 |  | 9 | 3.1 |  |  |  |
| Other | 81 | 32.8 | 4 | 25.0 |  | 56 | 19.1 | 3 | 27.3 |  |
